# Child health and human development over the lifespan

**DOI:** 10.3389/fpubh.2013.00001

**Published:** 2013-03-19

**Authors:** Joav Merrick

**Affiliations:** ^1^National Institute of Child Health and Human DevelopmentJerusalem, Israel; ^2^Office of the Medical Director, Health Services, Division for Intellectual and Developmental Disabilities, Ministry of Social Affairs and Social ServicesJerusalem, Israel; ^3^Division of Pediatrics, Hadassah Hebrew University Medical Center, Mt. Scopus CampusJerusalem, Israel; ^4^Kentucky Children's Hospital, University of KentuckyLexington, KY, USA

The topic of child health and human development is a wide area of interest spanning from pregnancy, delivery, childhood, adolescence, adulthood, and end of life. A study of health, development, and well-being over the lifespan.

Before birth through young adulthood there is a wide range of health issues that affect our children, such as general childhood illnesses, eating and obesity, accidents and injuries, and particular stages of life, such as teenage independence. Childs health and pediatrics focus on the well-being of children from conception through adolescence, but human development is a life span issue, so research in childhood does not stop with the end of adolescence, but we need a long-term and lifelong study to observe and understand the development process. Pediatrics is vitally concerned with all aspects of children's growth and development and with the unique opportunity that each child has to achieve their full potential as a healthy adult.

Pediatrics or child health was once not a specific entity, just as adolescence really did not exist as a concept, since all was a part of adult medicine. This field emerged in the nineteenth and early twentieth century as a medical specialty, because of the gradual awareness that the health problems of children were different from those of adults and children's response to illness, medications, and the environment is very depending upon the age of the child.

This uniqueness of children, along with diseases that are particular to this age group, has been responsible for the development of pediatrics as a specialty and for the creation of children's hospitals for the care of children.

## Child health research

These same factors have also driven the creation of child health research, but we are still only able to do a few large lifelong studies to see the effects of pregnancy or early childhood on health and well-being in adulthood and older age. Long-term birth cohort studies have been and are conducted in the United Kingdom under the auspices of the Centre for Longitudinal Studies in London, like the National Survey of Health and Development (NSHD) established in 1946, the National Child Development Study (NCDS) established in 1958, the 1970 British Cohort Study (BCS70), and the Millennium Cohort Study (MCS) established in 2000 (1). In Denmark with the Copenhagen Perinatal Birth Cohort of 9125 individuals born 1959–1961 at the maternity departments of the Copenhagen University Hospital, Rigshospitalet (2) and the Danish National Birth Cohort 1996–2002 of 101,042 pregnant women recruited in first trimester at first antenatal visit at the general practitioner with 96,986 children resulting from the pregnancies (3). In the United States the National Institute of Child Health and Human Development has recently also initiated a large prospective life-history study, the National Children's Study, examining the effects of the environment and genetics on the growth, development, and health of children with more than 100,000 children who will be followed up from conception to age 21 years (4).

Such cohort studies of child health and human development over the lifespan are very important for our understanding of trends in health and well-being, quality of life, and quality of care, which will reveal emerging of “new morbidities” as we have seen over the past 50 years in pediatrics (5), but such cohort studies are very expensive, huge logistics involved and not always possible to conduct.

## Growth and development

A healthy development begins before conception with parental health and their genetic composition and continues on to conception and through the prenatal period. Once delivered, new issues emerge, such as breastfeeding, newborn screening tests, health care appointments, and immunizations. Development constitutes a continuum and a child changes amazingly during the neonatal, newborn period, and early infancy. During this period there are many challenges both for the child, the parents, and the family and before you know it the child enter adolescence and adulthood.

## Current issues

CS Mott Children's Hospital at the University of Michigan in Ann Arbor conducts a National Poll on Children's Health in order to monitor the future. In their collaboration with Knowledge Networks in this nationally representative household survey they administer to a randomly selected, group of adult with and without children of about 2000 person that closely resembles the United States population. In 2010, the following overall health concerns for US children in 2010 and the percentage of adults who rate each as a “big problem” included (6):

- Childhood obesity, 38%
- Drug abuse, 30%
- Smoking, 29%
- Internet safety, 25%
- Stress, 24%
- Bullying, 23%
- Teen pregnancy, 23%
- Child abuse and neglect, 21%
- Alcohol abuse, 20%
- Not enough opportunities for physical activity, 20%
- Chemicals in the environment, 18%
- Sexting, 16%
- Depression, 15%
- Sexually transmitted infections, 15%
- School violence, 13%
- Asthma, 10%
- Neighborhood safety, 8%
- Autism, 8%
- Suicide, 8%

But the perception of the parent does not always portray the view of the child and researchers have therefore become concerned with the children's own perception of health. One study from Portugal (7) used creative drawing language to identify external factors perceived as negative or positive to health by children. The sample consisted of 130 children in 3rd and 4th classes from four randomly selected schools found that children value healthy food, physical activity, mental health, prevention of inappropriate substance consumption and health and environment. The drawings and comments showed links between diet and physical exercise, and between mental health and interpersonal relationships (7).

## Conclusions

Just a few decades ago, children born with significant congenital anomalies or genetic and metabolic diseases perished at an early age and very few survived into their teens and even less into adulthood. Congenital heart disease, major errors in metabolism, cancer, cystic fibrosis, and many other major diseases were fatal. Because of that many physicians in adult primary care did not have the opportunity to see patients with these problems and thus were unable to learn how to care for them.

With major advancements in medical knowledge, technology, imaging techniques, surgical skills, and pharmaceutical products as well as prosthetic devices, many of these patients now live much longer life and sometimes even close to the average life expectancy for the country at least in the developed world. With that, a new medical care challenge has been created and we have to take a life span approach.

In the *Frontier of Child Health and Human Development* we would like to provide an academic focal point for the scholarly interdisciplinary study of child life, health, public health, welfare, disability, rehabilitation, intellectual disability, and related aspects of human development over the life span. Research, clinical work, public service activities in the field of child health and human development over the life span will be important topics for this journal.
